# Malonate is relevant to the lung environment and induces genome-wide stress responses in Pseudomonas aeruginosa

**DOI:** 10.21203/rs.3.rs-4870062/v1

**Published:** 2024-09-10

**Authors:** Karishma Bisht, Moamen M. Elmassry, Hafij Al Mahmud, Shubhra Bhattacharjee, Amrika Deonarine, Caroline Black, Michael J. San Francisco, Abdul N. Hamood, Catherine A. Wakeman

**Affiliations:** Princeton University; Princeton University; Texas Tech University; Texas Tech University; Texas Tech University; Texas Tech University; Texas Tech University; Texas Tech University Health Sciences Center; Texas Tech University

**Keywords:** Pseudomonas aeruginosa, carbon metabolism, malonate, glycerol, stress response, metal homeostasis, aggregates

## Abstract

Versatility in carbon source utilization is a major contributor to niche adaptation in *Pseudomonas aeruginosa*. Malonate is among the abundant carbon sources in the lung airways, yet it is understudied. Recently, we characterized how malonate impacts quorum sensing regulation, antibiotic resistance, and virulence factor production in *P*. *aeruginosa*. Herein, we show that malonate as a carbon source supports more robust growth in comparison to glycerol in several cystic fibrosis isolates of *P. aeruginosa*. Furthermore, we show phenotypic responses to malonate were conserved among clinical strains, i.e., formation of biomineralized biofilm-like aggregates, increased tolerance to kanamycin, and increased susceptibility to norfloxacin. Moreover, we explored transcriptional adaptations of *P*. *aeruginosa* UCBPP-PA14 (PA14) in response to malonate versus glycerol as a sole carbon source using transcriptomics. Malonate utilization activated glyoxylate and methylcitrate cycles and induced several stress responses, including oxidative, anaerobic, and metal stress responses associated with increases in intracellular aluminum and strontium. We identified several genes that were required for optimal growth of *P. aeruginosa* in malonate. Our findings reveal important remodeling of *P. aeruginosa* gene expression during its growth on malonate as a sole carbon source that is accompanied by several important phenotypic changes. These findings add to the accumulating literature highlighting the role of different carbon sources in the physiology of *P. aeruginosa* and its niche adaptation.

## Introduction

*Pseudomonas aeruginosa* is an opportunistic pathogen that infects various hosts including humans [1]. It can infect a wide range of body sites, e.g., lungs in cystic fibrosis patients, wounds and blood circulation in burn and trauma patients [2]. Its versatile metabolism enables this bacterium to survive different environmental conditions by utilizing a multitude of carbon sources [3]. This metabolic versatility is controlled by complex regulatory networks [4]. The utilization of certain carbon sources is linked to antibiotic tolerance and altered virulence of *P. aeruginosa* [5–7]. Furthermore, the activation of metabolic networks by specific carbon sources involves a stress response depending on the environmental conditions [8, 9]. However, the link between carbon source utilization and stress response is not clear.

Glycerol is a byproduct of phosphatidylcholine, a major lung surfactant. Glycerol is recognized as a key carbon source for *P. aeruginosa* and has been included in *Pseudomonas* Isolation Agar (PIA), a traditional lab medium used for selective growth of the bacterium in which glycerol serves as a carbon source and enhances pyocyanin production. Yet, it is unknown if other available carbon sources in the lung may be advantageous for the growth of *P. aeruginosa*. One example is malonate, a naturally occurring organic acid, which is utilized by several microorganisms as a carbon source [6, 10–13]. Previous studies suggest the potential clinical importance of malonate. For example, malonate utilization genes are overexpressed in *P. aeruginosa* grown ex vivo in blood from trauma patients [6]. Moreover, malonate increases the tolerance of *P. aeruginosa* to aminoglycoside antibiotics and influences its quorum sensing circuit and virulence factors [5, 6, 14]. These malonate-induced shifts in metabolism were also associated with the production of surface-free biofilm-like aggregates (also known as flocs) embedded in a biomineralized matrix [14]. Similar bacterial aggregates have been associated with biofilms and lung infections [15, 16]. However, it is not known how carbon source utilization is involved in this phenotype.

Herein, we aim to focus on the clinical relevance of malonate if it can support the growth of cystic fibrosis (CF) isolates of *P. aeruginosa* as a sole carbon source. Furthermore, we study the transcriptional regulation occurring in *P. aeruginosa* during malonate utilization. Our findings highlight the lifestyle modulation effect of carbon sources on *P. aeruginosa* and provide evidence of malonate-induced stress response and its associated phenotypes in this pathogen.

## Results

### Malonate is abundant in the human airways and promotes the growth of P. aeruginosa cystic fibrosis isolates

**Figure 1:**
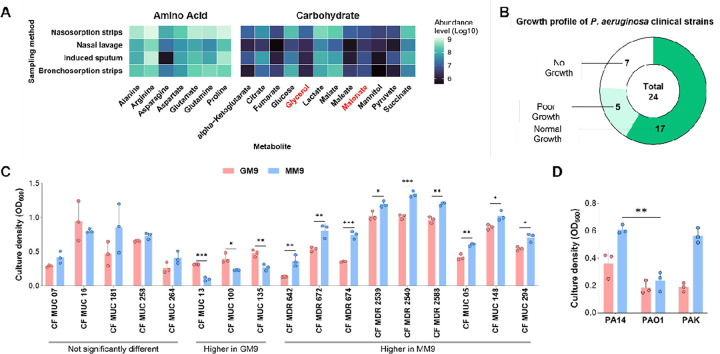
Malonate is an airway-abundant carbon source that can be broadly utilized by many *P. aeruginosa* strains. **(A)** Abundance of metabolites detected and confirmed using chemical standards. Metabolites were measured in airway samples using the four sampling methods used by Farne et al. Data were extracted from a publicly available dataset (Farne et al. 2017). **(B)** Proportion of clinical strains that robustly grew in GM9 and MM9 (Normal growth), poorly grew (OD_600_ below average, 0.5), or did not grew (No growth). **(C)** Culture density of clinical strains based on OD_600_ measurement after 16 hours of incubation at 37°C. **(D)** Culture density of lab strains based on OD_600_ measurement after 16 hours of incubation at 37°C. CF: cystic fibrosis, MUC: mucoid strains, MDR: multidrug-resistant strain. Values represent the means of three independent experiments ± 1 standard deviation. Statistical significance was calculated by two-tailed unpaired t-test. * P < 0.05, ** P < 0.01, *** P < 0.001, and **** P < 0.0001.

The human airway is a complex environment with a multitude of metabolites that can serve as a carbon source for *P. aeruginosa*. Glycerol, a degradation product of the major lung surfactant phosphatidylcholine, has been recognized as a key carbon source for *P. aeruginosa*. Furthermore, glycerol has been included in *Pseudomonas* Isolation Agar (PIA), a traditional lab medium selective growth of *P. aeruginosa*, in which glycerol serves as a carbon source and enhances pyocyanin production. However, many metabolites are present in the lung and may serve as carbon sources for *P. aeruginosa*, e.g., succinate (Fig. 1A) [17]. Unlike glycerol, malonate has not been well-studied, and whether it can support the growth of *P. aeruginosa* cystic fibrosis (CF) isolates is still unknown.

To this end, we used a library of 24 clinical isolates of *P. aeruginosa* (Table 1) to compare their growth in M9, a minimal medium, containing malonate (MM9) as a sole carbon source vs. glycerol as a control (GM9). While the majority (17 isolates) were able to grow in GM9 and MM9, five isolates grew poorly, and two showed no growth (Fig. 1B). Only three isolates showed higher growth in GM9 vs. MM9 (Fig. 1C). Surprisingly, more than half of the clinical isolates showed more robust growth in MM9 rather than in GM9 (Fig. 1C). This was the same observation with *P. aeruginosa* strain UCBPP-PA14 (PA14), a commonly used lab strain, as well as other lab strains (Fig. 1D, Table 2).

### Conservation of malonate-associated phenotypes across clinical and lab strains

**Figure 2:**
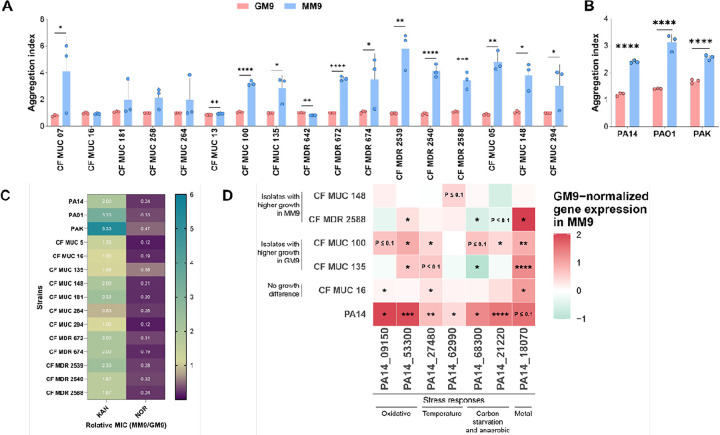
Phenotypes of *P. aeruginosa* clinical lab and strains grown in MM9 and GM9. **(A-B)** Aggregation index was calculated as the ratio of OD_600_ post-sonication to OD_600_ pre-sonication of bacterial culture. **(C)** Minimum inhibitory concentration (MIC) of KAN and NOR against *P. aeruginosa* strains in MM9 vs. GM9. The MIC values for KAN and NOR in MM9 and GM9 were determined through a broth microdilution assay, and the MIC values for each antibiotic in MM9 were normalized to the corresponding antibiotic in the GM9 medium. **(D)** Gene expression of selected stress responsive genes in clinical strains. PA14 and the clinical strains were grown overnight in GM9 and MM9 before their RNA was extracted, followed by qRT-PCR. Expression values were normalized to a reference housekeeping gene, i.e., the 16S ribosomal RNA gene *PA14_08570*. Shown expression values in MM9 were normalized to that of GM9. Color indicates the average of biological triplicates. CF: cystic fibrosis, MUC: mucoid strains, MDR: multidrug-resistant strain, KAN: Kanamycin, NOR: Norfloxacin. Values represent the means of three independent experiments ± 1 standard deviation. Statistical significance was calculated by two-tailed unpaired t-test. * P < 0.05, ** P < 0.01, *** P < 0.001, and **** P < 0.0001.

Previously, we observed strong phenotypes for PA14 grown in MM9, i.e., aggregation and differential susceptibility to antibiotics [14]. Therefore, we wanted to test if these phenotypes are conserved across clinical and lab strains. First, we examined if the bacterial culture of *P. aeruginosa* can form aggregates in MM9 versus GM9 media. We found that aggregation is a common outcome of malonate-utilization among the isolates (around two-thirds showed high aggregation index) (Fig. 2A). Additionally, all lab strains showed aggregation in MM9 (Fig. 2B).

Next, we wanted to examine if both clinical and lab strains exhibited increased tolerance to kanamycin and greater susceptibility to norfloxacin in MM9 compared to GM9 [18, 19]. We examined the impact of malonate and glycerol as carbon sources on antibiotic susceptibility using a broth microdilution assay for both kanamycin (up to 1200 μg/ml) and norfloxacin (up to 100 μg/ml). In line with the lab strains, most clinical isolates (CF MUC 5, CF MUC 148, CF MUC 181, CF MUC 264, CF MDR 672, CF MDR 674, CF MDR 2539, CF MDR 2540, and CF MDR 2588) exhibited increased tolerance to kanamycin in MM9 compared to GM9 (Fig. 2C and Table 3). However, unlike control strains, CF MUC 13 and CF MUC 100 displayed heightened kanamycin tolerance in GM9 compared to MM9. Furthermore, several tested strains maintained consistent susceptibility or tolerance to kanamycin regardless of the carbon source. CF MUC 7 did not grow in MM9 but thrived in GM9. While we did observe growth for CF MUC7 in MM9 in shaking growth conditions as shown previously, we believe that a static growth set up could have resulted for this no growth phenotype. Interestingly, CF MUC 7 did not grow in GM9 when glacial acetic acid was used as a vehicle control. In addition, all strains demonstrated greater tolerance to norfloxacin in GM9 media than MM9 (Fig. 2C and Table 3). In summary, our results indicate that both lab and clinical *P. aeruginosa* strains tend to exhibit increased tolerance to kanamycin and heightened susceptibility to norfloxacin when malonate is utilized as a sole carbon source. Overall, these results suggest that while some phenotypes associated with malonate utilization are conserved across clinical and lab strains, others are strain specific.

### Transcriptional activation of the glyoxylate and methylcitrate cycles by malonate

Motivated by the phenotypes observed in the presence of malonate in comparison to glycerol, we aimed to decipher how malonate utilization alters *P. aeruginosa* at the transcriptional level. We examined the transcriptomic changes in PA14 when grown in MM9 vs. GM9. Transcriptomic data analysis was performed using Rockhopper 2 [20]. Overall, 2,478 genes were differentially expressed (Benjamini-Hochberg procedure-adjusted P-value ≤ 0.05). In comparison to GM9, MM9 induced the expression of 1,370 genes and downregulated the expression of 1,108 genes. We validated our transcriptomic results, obtained from RNA-seq analysis, by examining the expression of a subset of genes of interest using qRT-PCR **(Supplementary Fig. 1)**.

The gene expression data enabled us to establish which metabolic pathways were activated by the growth of PA14 on malonate as a sole carbon source. As expected, the growth of *P. aeruginosa* in minimal medium with only malonate supplemented as a sole carbon source upregulated the expression of the 10 genes involved in malonate utilization, i.e., regulation, uptake, and decarboxylation **(Supplementary Fig. 2)**. The average expression ratio of these malonate utilization-related genes was 135 times higher in MM9 than in GM9.

**Figure 3:**
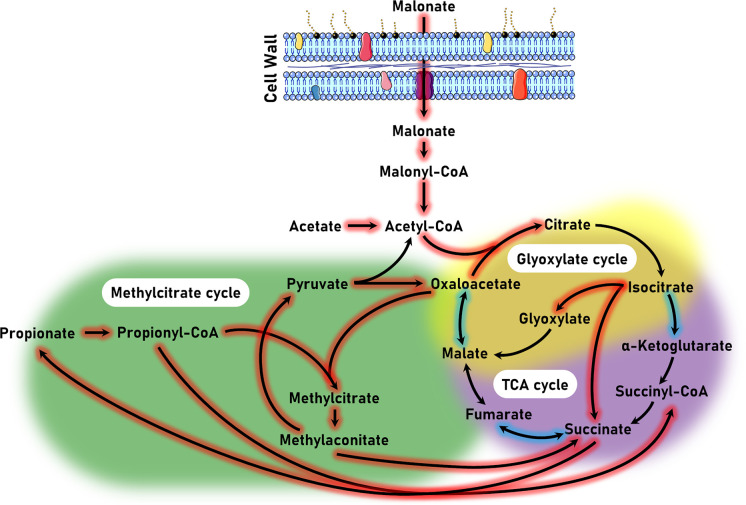
Transcriptomic analysis revealed the differential regulation of genes associated with different metabolic cycles. Central carbon metabolic pathways involved in malonate utilization in *P. aeruginosa*. Arrows represent the differentially expressed genes and their corresponding reactions. Red-highlighted arrows indicate upregulation and blue highlighting indicates downregulation. The three main metabolic cycles (i.e., TCA, glyoxylate, and methylcitrate) are colored as well.

Next, we identified 534 differentially expressed genes mapped to KEGG metabolic pathways, of which 70 mapped to carbon metabolism. Specifically, over 50 genes were mapped to pathways involving tricarboxylic acid (TCA), glyoxylate, and methylcitrate cycles (Fig. 3). Upon utilization of malonate, acetyl-CoA is produced, which then feeds carbon metabolic circuits. Unlike the inhibited TCA cycle genes, the genes of glyoxylate and methylcitrate cycles were activated upon malonate utilization. These findings provide insight into how malonate utilization regulates different carbon metabolic pathways which could be a crucial strategy for its adaptation in different niches.

### Stress response is induced by malonate-utilization

Next, we investigated whether malonate utilization is linked to eliciting stress response in *P. aeruginosa*. Surprisingly, we identified over a hundred genes that are differentially expressed and linked to various stress responses. These can be broadly grouped as metal stress response (Table 4), oxidative stress response (Table 5), or carbon starvation/anaerobic stress response (Table 6), and a few general stress response genes that we grouped into a temperature, osmolarity, and pH stress response category (Table 7).

### Metals and oxidative stress response

Our transcriptomic analysis revealed that malonate utilization relative to glycerol utilization induces genes that are associated with copper toxicity response in PA14 (Table 4) [21]. Genes representing the copper resistance regulon include the operon consisting of the five genes *PA14_18810–18860* that were upregulated an average of 103.5 times in MM9 relative to GM9. Two copper-related genes, *PA14_18800* and PA14_18070, were induced 7.2 and 46.8 times, respectively. These genes code for proteins with a copper chaperone CopZ domain. Three genes *PA14_18760–18790* coding for a homolog of the resistance-nodulation-division (RND) efflux pump MexPQ-OpmE were also induced with an average of 15.6 times. This efflux pump was observed to be activated by copper [22]. *PA14_13170*, a *copA1/cueA* homolog (a P-type ATPase), was induced 21.3 times. These copper resistance-regulated genes are known to be controlled by the transcriptional regulator CueR, and mutations in any of these genes result in a higher sensitivity to copper [21]. Surprisingly, this gene’s homolog (*PA14_63170*) was not differentially expressed (P-value = 0.2). Several other genes related to metal stress response were differentially regulated with malonate utilization. For example, the four genes coding for the RND efflux pump, MexGHI-OpmD, were upregulated with an average of 23.5 times. Other genes included those coding for cation-transporting P-type ATPases (Table 4). Lastly, *PA14_65000* that codes for azurin was upregulated 3.7 times. We also observed that the genes involved in pyoverdine biosynthesis and uptake were downregulated in MM9, suggesting that the cell is shutting down its metal import systems (Table 4). The *pvdD* gene was downregulated 3 times, while the pyoverdine biosynthesis protein PvdE was downregulated 5 times. These results suggest that malonate utilization results in metal stress response due to either the accumulation of metals accompanied by malonate utilization or an alteration in the regulatory network controlling metals sensing independent of metal concentration.

Because oxidative stress is strongly linked to metal stress, we aimed next to investigate if oxidative stress-related genes were also differentially regulated by malonate utilization [23, 24]. Indeed, over a dozen genes involved in oxidative response were upregulated (Table 5). Catalase-coding genes (*katA* and *katB*) were upregulated 54.5 and 34.4 times, but *katE* was downregulated 3.1 times. *PA14_61020* is a gene in the same operon as *katB* was found to be upregulated 11.1 times. Its gene product has an ankyrin repeat domain, which plays a role in signal transduction [25]. *PA14_21530* is another gene harboring ankyrin repeat domain in its protein product that was significantly upregulated 112.2 times. Interestingly, the expression of this gene is positively regulated by MvfR [26] and associated with oxidative stress adaptation [27]. *PA14_22320* is another gene that was significantly upregulated 184.6 times. It is possibly controlled by MvfR [26], and its expression is induced by hydroxyl radicals [28]. *PA14_03090* was another gene that was upregulated, and evidence suggests that it is required for hydrogen peroxide resistance [27]. Three alkyl hydroperoxide reductase-coding genes (*ahpB, ahpC,* and *ahpF*) were upregulated with an average of 76.3 times—among them, *ahpB* differential expression was the highest, 171.1 times. Five peroxidase-coding genes were upregulated such as glutathione peroxidase, thioredoxin reductase 2, and cytochrome c551 peroxidase. These results are consistent with our previous finding where we observed an increase in the catalase activity of PA14 grown in MM9 [14].

Pyomelanin is a pigment produced by *P. aeruginosa*, and one of its main functions is to provide protection against oxidative stress [29]. Our results suggest that malonate utilization is associated with its upregulation (Table 5). Genes *PA14_52990* and *PA14_53070*, which are involved in the biosynthesis of homogentisate (the precursor of pyomelanin) from chorismate were upregulated. Moreover, genes (*PA14_57830, PA14_57850,* and *PA14_57870*) involved in the transport of homogentisate [30] and possibly the extracellular accumulation of pyomelanin were upregulated as well. Overall, these results suggest that malonate utilization in *P. aeruginosa* is accompanied by an oxidative stress response.

### Carbon starvation and anaerobic stress response

Just as metal stress and oxidative stress are inseparably linked, carbon starvation and anaerobic stress response share many facets. Transcriptomic analysis of *P. aeruginosa* during malonate utilization reveals that *P. aeruginosa* is undergoing carbon starvation and anaerobic stress response (Table 6). The expression of *rpoS*, which encodes a sigma-factor known to be associated with carbon or oxygen limitation [31], was upregulated 3.5 times. The expression of *psrA*, an *rpoS* transcriptional regulator [32], was also upregulated 1.6 times. The *aa*_3_-type cytochrome *c* oxidase, encoded by the *coxBA-PA14_01310-coIII* gene cluster and induced by RpoS upon carbon limitation [3], was also found to be upregulated an average of 4.4 times.

All 13 genes (*nuoN, nuoM, nuoL, nuoK, nuoJ, nuoI, nuoH, nuoG, nuoF, nuoE, nuoD, nuoB,* and *nuoA*) constituting the *nuo* operon were upregulated on average 3.3 times (Table 6). This operon codes for the NADH dehydrogenase (NADH: quinone oxidoreductase). This enzyme translocates protons, oxidizes NADH to NAD^+^ [33], and is required for anaerobic growth [34]. Furthermore, this system is important for *P. aeruginosa* virulence and provides resistance to aminoglycoside antibiotics [35].

We also identified a putative ABC transporter encoded by *PA14_69090, PA14_69070,* and *PA14_69060* that was upregulated ~ 3 times. A previous study suggests that this ABC transporter is also required during oxygen limitation [32]. The expression of the outer-membrane protein encoded by *oprG*, was upregulated 31 times. OprG is a specific transporter of hydrophobic molecules and its expression is induced under anaerobic conditions [36].

Five genes encoding proteins with the universal stress protein family domains were upregulated (Table 6). These included *uspO, uspK, uspN, uspL,* and *uspM* with an average increase of 18.7 times. These genes play a role in response to different stressors—more importantly, anaerobic stress conditions or oxygen limitation [37]. All genes of *arcDABC* operon, which is responsible for arginine fermentation, were upregulated. The expression of *uspK, uspN* and *arcDABC* was reported to be induced in biofilms or anaerobic environments [38]. Two genes (acka and pta) involved in pyruvate fermentation and anaerobic metabolism [39] were also upregulated. Moreover, the *cbb*_3_-type cytochrome *c* oxidase, encoded by *PA14_44340* and *PA14_44350*, was also upregulated. This system is induced under an oxygen limited environment [40]. Lastly, another recently identified gene that is associated with survival under low oxygen conditions, *mhr*, was found to be upregulated almost 27 times [41].

The *dnr* gene (*PA14_06870*) encoding the transcriptional regulator Dnr was upregulated 9 times (Table 6). Dnr senses nitric oxide [42] and regulates the denitrification gene [43]. During the denitrification process, nitrate is used as an alternative terminal electron acceptor and reduced to nitrogen [44]. Our results showed that 20 genes that are part of the denitrification operons (*nar, nir, nap, nor,* and *nos*) were induced. Our finding of increased expression of many *nos, nor, nir, and dnr* genes by cells suggests that *P. aeruginosa* is exposed to anaerobic conditions when grown in malonate as a sole carbon source. The *fhp* gene, and its transcriptional regulator *fhpR*, were upregulated as well. This flavohemoglobin system is nitric oxide-responsive and impairs the dispersal response to nitric oxide [45]. These results suggest that nitric oxide is produced during malonate utilization, thus eliciting an anaerobic (low oxygen) stress response. This was supported by the upregulation in the three genes constituting *hcn* operon that is responsible for the synthesis of hydrogen cyanide, which is induced under hypoxic environment [46]. Moreover, the recently characterized small RNA, *sicX*, encoded by PA14_46160 was upregulated ~ 6 times. It is worth noting that *sicX* was found to be induced by low oxygen and is important in the transition between chronic and acute infection stages in *P. aeruginosa* [47].

Eight genes involved in the biosynthesis of pyocyanin were significantly upregulated (Table 6). For example, *phzB*_*1*_, one of the main biosynthetic operons *phz*_*1*_, was upregulated 109.5 times. But four genes of the redundant operon *phz*_*2*_ were downregulated. Analysis of the differential regulation of these biosynthetic operons, though note-worthy, is beyond the scope of this study. This possible increase in pyocyanin fits with our hypothesis, as pyocyanin is important for *P. aeruginosa* to maintain redox homeostasis and survive in biofilms under oxygen-depleted environments [3, 48]. The upregulation of the phenazine biosynthesis genes is further supported by the upregulation in biosynthetic genes (*phnAB* and *pqs* operon) of *Pseudomonas* quinolone signal (PQS), which tightly regulates pyocyanin production. Also, our data is consistent with our prior report that showed that growth in MM9 impacts pyocyanin production and quorum-sensing regulated pathways [14]. *P. aeruginosa* has multiple resistance mechanisms to protect itself from the toxic effects of pyocyanin and maintain redox homeostasis [49]. This was observed through the upregulation of expression of genes encoding the monooxygenase PumA and RNA efflux pump MexGHI-OpmG. One operon consisting of two genes associated with cyanide resistance (i.e., *PA14_10550* and *PA14_10560*) was upregulated [50, 51]. Besides this operon, a thiosulfate sulfur-transferase encoded by *PA14_30430* was upregulated. Although this is not experimentally confirmed yet, we hypothesize that the thiosulfate sulfur-transferase (encoded by *PA14_30430*), which carries repeated rhodanese homology domains, detoxifies cyanide to sulfite and thiocyanate. Then, the sulfite reductase (encoded by *PA14_10550*) reduces sulfite to sulfide. Whether the hypothetical protein encoded by *PA14_10560* is possibly involved is yet to be determined. Overall, the results suggest that malonate utilization is tightly linked with anaerobic respiration and associated with the carbon starvation stress response.

### Temperature, osmolarity, and pH stress response

Even though all cultures were incubated at 37°C, malonate utilization was associated with heat-shock response on the transcriptomic level (Table 7). This was accompanied by an increase in the expression of four heat-shock genes encoding for proteins (IbpA, HtpX, HtpG, and GrpE) with an average of 5.6 times and decrease in the expression of two cold-shock proteins with an average of 4.6 times. Our results are consistent with the findings of other groups that indicated heat-shock responses can be induced by conditions that did not alter temperature such as alkaline pH or carbon starvation [52–54]. Finally, we observed the upregulation of certain genes involved in osmolarity and pH fluctuations (Table 7). For example, the expression of the transcriptional regulator OmpR was significantly upregulated. Among its regulated genes are *htpX* (membrane protease), *PA14_72930* (predicted lipid-binding transport protein, Tim44 family), and *PA14_30410* (YccA-like protein). All of *ompR, htpX, PA14_72930,* and *PA14_30410* protect *P. aeruginosa* against osmolarity and pH stressors [55]. The role of these genes involves maintaining membrane integrity. Because of this critical role, the function of those genes involves the protection against aminoglycoside antibiotics [55]. Overall, these results suggest that malonate utilization induces a global stress response in *P. aeruginosa* that spans various physical and physiological stressors. However, it is not clear what these adaptations are responsive to exactly, which we aimed to address next.

### Gene expression in CF isolates

After observing that many of the clinical isolates show similar phenotypes to that of PA14, we wondered if we could also observe similar trends at the transcriptional level. To evaluate this, we selected five clinical strains that showed variable growth phenotypes in MM9 and GM9 and examined their expression of stress response genes. Briefly, MUC 16 was selected as it showed no significant growth difference in MM9 and GM9, MDR 2588 and MUC 148 showed higher growth in MM9, and MUC 135 and MUC 100 showed higher growth in GM9. We examined the expression of genes related to stress response that we identified to be upregulated in MM9. These included genes belonging to metal, oxidative, anaerobic, and temperature stress responses. While we observed a significant variability in the gene expression of the selected genes, a few showed similar trends (Fig. 2D). Specifically, genes involved in metal and oxidative stress response (*PA14_53300* and *PA14_18070*) were consistently upregulated in MM9 vs. GM9 in four out of the five selected clinical isolates, regardless of their growth preference (Fig. 2D), like what was previously observed in PA14. In general, malonate utilization appears to be linked to comparable stress response gene expression patterns and phenotypes among numerous clinical isolates and laboratory strains of *P. aeruginosa*.

### Aluminum is accumulated intracellularly in the presence of malonate

**Figure 4:**
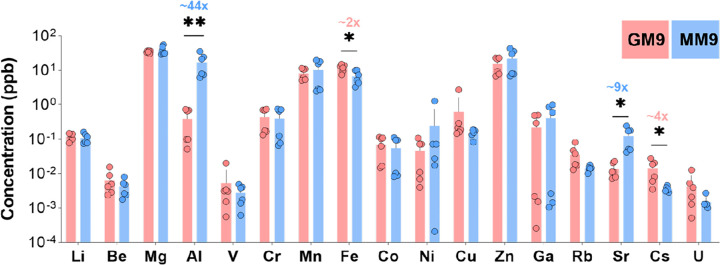
Effect of malonate utilization on accumulation of metal ions in *P. aeruginosa*. **(A)** Accumulation level of all measured metal ions using ICP-MS. PA14 was grown in Chelex-treated malonate and glycerol supplemented Chelex-treated M9. Samples were processed for ICP-MS analysis as mentioned in the Experimental Procedures. Samples were normalized based on protein content, then analyzed for the accumulation of the metal ions using ICP-MS. Error bars represent 1 standard deviation of the results from triplicate samples. Unpaired t-test (two-tailed) was used to measure statistical significance. * P < 0.05 and ** P < 0.01. The concentration of metal ions was measured in parts per billion (ppb). This experiment was repeated three times on three different days and repeated once using the previously used carbon source concentrations and once using equimolar concentrations.

Because the transcriptomic analysis revealed a striking metal stress response, we hypothesized that malonate utilization results in the accumulation of metal ions within the bacterial cells. To determine the effect of malonate or glycerol as sole carbon sources on the accumulation of various metal ions within *P. aeruginosa* cells, we used inductively coupled plasma mass spectrometry (ICP-MS) to detect the level of metal ions. Because chemicals may carry metal impurities, we used Chelex-treated M9 and Chelex-treated carbon sources. Chelex is a chelating agent that binds polyvalent metal ions; thus, this treatment removed the possibility that metal changes in the different treatments could be driven by trace metal contaminates in the different carbon sources. This procedure eliminated several metals essential for growth. Therefore, to complement those metals, we added a cocktail of these essential metal ions to the prepared media (i.e., Mg 1mM, Mn 25 μM, Ca 100 μM, Fe 5 μM, Zn 25 μM, Co 0.1 μM, and Cu 0.1 μM). After overnight incubation of *P. aeruginosa* in Chelex-treated GM9 and MM9, cells were collected and then prepared using an established protocol following slight modifications [56]. Using ICP-MS analysis of the overnight cultures, we measured the concentration of 17 metal ions (Fig. 4). While most of the measured metal ions were similarly abundant in cells obtained from GM9 and MM9, two metal ions (i.e., Al and Sr) showed a statistically significant difference between MM9 and GM9. Specifically, Al and Sr were ~ 44 and ~ 9 times, respectively, more abundant intracellularly in MM9 versus GM9 (Fig. 4). The mechanism by which malonate utilization induces the accumulation of metal ions is not clear yet. One possible explanation might be attributed to the lower level of pyoverdine in MM9 since this molecule can decrease toxic metal accumulation [57, 58]. Thus, a decrease in pyoverdine production might have enabled this metal accumulation in MM9.

Because we observed an upregulation in all known regulons related to copper stress response, we expected to find Cu to be accumulated in MM9. Surprisingly, this was not the case (Fig. 4). One possible explanation is that while Cu gets accumulated at first, *P. aeruginosa* then adapts to get rid of excess Cu through the production of a secreted copper-containing small molecule named fluopsin C [59]. This hypothesis is strengthened by the prominent upregulation (over 100 times higher in MM9 vs. GM9) of the biosynthetic operon consisting of five genes *PA14_18810–18860* that is responsible for fluopsin C biosynthesis.

Motivated by the phenotype of *P. aeruginosa*, in which its malonate utilization induces the expression of biosynthetic genes of fluopsin C and pyocyanin, and knowing both of these molecules act as antibiotics against *Staphylococcus aureus* [59, 60], we hypothesized that this could benefit *P. aeruginosa* in its competition against other bacteria it may encounter in the lung environment. We further hypothesized that the survival of *S. aureus* will decrease when co-cultured with *P. aeruginosa* in MM9, but not in GM9. To test this hypothesis, we used *S. aureus* strain JE2, an established methicillin-resistant lab strain [61], to study its survival in the presence of *P. aeruginosa* in MM9 and GM9. A co-culture of *P. aeruginosa* and *S. aureus* in a 1:1 ratio was grown in both MM9 and GM9 overnight. Then, cells were serially diluted and plated on selective media. First, we confirmed that no difference was observed in the growth of *P. aeruginosa* when co-cultured with *S. aureus* **(Supplementary Fig. 3A).** In contrast, we observed a significant reduction in the number of *S. aureus* cells when co-cultured with *P. aeruginosa* in MM9, while no such difference was observed when co-cultured with *P. aeruginosa* in GM9 **(Supplementary Fig. 3B).** While the higher levels of pyocyanin and possibly fluopsin C in MM9 as compared to GM9 may explain the increased death rate of *S. aureus* in MM9, other virulence or metabolic factors may contribute to this phenotype as well.

### Genetic requirements for P. aeruginosa to grow using malonate as a sole carbon source

**Figure 5:**
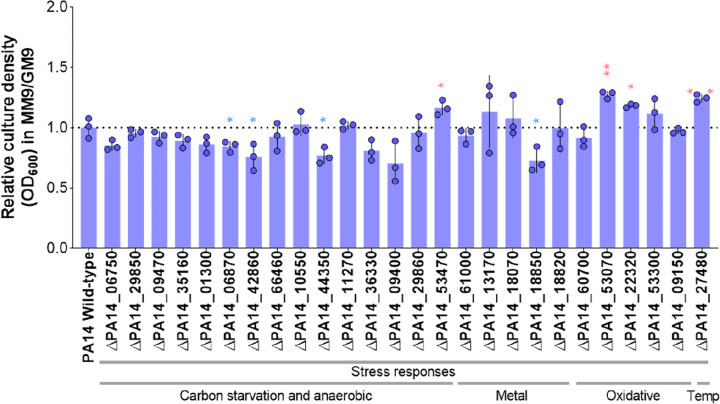
The growth of different *P. aeruginosa* strains that carry mutations in genes involved in stress adaptation. Culture density ratio of each strain grown in MM9 relative to GM9 culture density. Bacteria were grown for 24 hours before measuring OD_600_. The mutants were run in three replicates, on three independent days. Bars represent the mean of three biological replicates. Error bars represent the standard deviation of the replicates. Data points were normalized to PA14 wild type mean. Unpaired t-test (two-tailed) was used to measure statistical significance. * P < 0.05 and ** P < 0.01. Statistical significance indicates statistical difference between the growth of the mutant vs. that of wild-type PA14. Temp: Temperature.

The observed upregulation in many stress-related genes raises an interesting question: Are these stress-related genes essential for the growth of *P. aeruginosa* in MM9 to cope with the stress induced by malonate utilization? To address this question, we performed a high-throughput screening on a commercially available transposon mutant library of *P. aeruginosa* UCBPP-PA14 containing over 5,500 unique mutants [62]. We grew wild-type PA14 and the mutants in GM9 and MM9 and measured bacterial growth using optical density (OD_600_). We observed a variable number of preliminary candidate mutants showing no growth in either MM9, GM9, or both **(Supplementary Table 1).** Upon the completion of the primary screening, we selected the hits of interest that overlapped with our transcriptomics dataset for further testing. We selected these mutants along with the wild-type (WT) PA14 to reassess their growth in MM9 and GM9 **(Supplementary Fig. 4A and 4B,** Fig. 5). While some mutants showed a statistically significant difference in their growth in comparison to that of WT, only Δ*PA14_27480* showed remarkable defective growth in both MM9 and GM9. The expression of *PA14_27480* was upregulated 8 times in MM9 vs. GM9 (Table 7). *PA14_27480* codes for the heat shock protein HtpX. HtpX and other heat-shock proteases allow *P. aeruginosa* to cope with protein misfolding stress induced by high temperature, carbon starvation, or alkaline pH, thus promoting its survival [54]. Eight mutants showed differential growth in MM9 or GM9, four of which showed more defective growth in MM9 rather than GM9 (Fig. 5). Δ*PA14_18850* caught our attention because its expression was ~ 150 times higher in MM9 vs. GM9. Furthermore, *PA14_18850* codes for FlcB, which catalyzes the first step in fluopsin C biosynthesis. Because fluopsin C plays a role in copper detoxification, this suggests that this functional adaptation is important for *P. aeruginosa* to cope with metal stress response in MM9.

## Discussion

Bacteria occupying different niches is often dependent on their carbon-source utilization versatility. This can in turn have a profound impact on their growth rate, virulence, phenotypes, and resistance to antibiotics [9, 63–66]. Earlier studies have described the role of carbon sources in the metabolism of *P. aeruginosa* [67–70]. Our study highlights the role of malonate as a sole carbon source in inducing a global stress response in *P. aeruginosa*.

We first asked if phenotypes associated with malonate-utilization were similar in lab and clinical strains of *P. aeruginosa*. Interestingly, most of the tested strains demonstrated better growth in MM9 vs. GM9. This result is of high clinical relevance because the traditional lab medium for selective growth of *P. aeruginosa* is *Pseudomonas* Isolation Agar (PIA), which requires glycerol to be added to it (2%) to serve as an energy source and to enhance pyocyanin production (BD Diagnostics or Remel). If strains of *P. aeruginosa* do not utilize glycerol as a carbon source, this can hinder isolating such strains that may utilize another carbon source such as malonate or mischaracterize *P. aeruginosa* as malonate is a much better enhancer of pyocyanin production than glycerol [14]. This is further supported by previous studies that investigated nutrient availability in sputum samples. For example, a study showed that malonate is among the abundant metabolites in the sputum of healthy volunteers [17]. However, the abundance of malonate in the sputum of cystic fibrosis patients is an important issue that warrants further investigation. Therefore, our results highlight the importance of studying the nutrient adaptation of different strains of *P. aeruginosa* to assist in their identification and isolation. Furthermore, we showed that the expression of certain genes involved in stress response are similarly overexpressed in clinical strains of *P. aeruginosa*. Overall, our findings show that clinical strains have unique transcriptional features that may deeply impact *P. aeruginosa*’s adaptation to specific environments found inside the host. Further genomic investigations are required on more clinical isolates to decipher potential mechanisms that facilitate their adaptation in response to different carbon sources. Further research on the metabolomics and proteomics analysis of *P. aeruginosa* exposed to malonate will provide us with valuable insights into the specific metabolites and proteins that are generated or triggered in the presence of this carbon source. Such pathways can eventually be used as a potential therapeutic target to combat *Pseudomonas*-associated infections and help us identify new strategies complementary or alternatives to antibiotics to efficaciously combat this notorious pathogen.

Our present findings align with the results obtained in our previous work, demonstrating increased and reduced tolerance to kanamycin and norfloxacin, respectively, in MM9 compared to GM9, for lab strains [18, 19]. In this latest investigation, we increased the spectrum of our lab strains and incorporated clinical strains to reinforce and validate our earlier observations. Most clinical strains, including the three lab control strains, exhibited heightened tolerance to kanamycin in MM9 compared to GM9. Conversely, all clinical strains and the three lab strains, demonstrated increased susceptibility to norfloxacin in MM9 as opposed to GM9.

Next, we wanted to investigate the transcriptional changes associated with this carbon source. It is known that the differential activation of the glyoxylate cycle in the presence of different carbon sources can affect both the pathogenesis and virulence of *P. aeruginosa* [71]. Our transcriptomic data pointed out that, in the presence of malonate, *P. aeruginosa* implements specific pathways to survive and adapt to its environment, modifying its virulence and stress response. In our study, we found that both the glyoxylate and methyl citrate cycle-associated genes were upregulated in the presence of malonate. The transcriptomic data further highlighted the intimate interaction between malonate utilization and various stress responsive genes in *P. aeruginosa*. Our transcriptomic data showed an upregulation in genes associated with metal stress response. This intrigued us to examine the metal ions accumulation in the presence of malonate. Aluminum and strontium were the two main metals that showed a significant accumulation in MM9 vs. GM9. We speculate that aluminum accumulation could be the result of differential siderophore production [72]. While we expected to observe copper accumulation in MM9, we observed none. We speculate that as an adaptation strategy, *P. aeruginosa* eliminates excess copper through the production and secretion of the copper-containing small molecule fluopsin C [59]. This is supported by the remarkable upregulation of the fluopsin C-biosynthetic genes.

Because *P. aeruginosa* and *S. aureus* are known to cause chronic infections, including lung infections, we were curious if malonate influences their interspecies interaction. A recent study demonstrated that the ability of *P. aeruginosa* to break down acetoin induces trophic cooperation between *S. aureus* and *P. aeruginosa* and improves their survival [73]. Our experiments revealed that malonate-utilization allowed *P. aeruginosa* to outcompete *S. aureus*. This could be explained by our previous observations showing that malonate induces the expression of biosynthetic genes of fluopsin C and pyocyanin, both of which are toxic to *S. aureus* [59, 60, 74]. We excluded the possibility that acetoin could be involved here because our transcriptomic data showed an upregulation in metabolic genes of acetoin, which should be improving *S. aureus* fitness, but was not observed here. However, other virulence factors not examined in our study could be involved too. This suggests that in a niche where malonate is available as a carbon source, it can allow *P. aeruginosa* to outcompete *S. aureus*.

Overall, we present malonate as an important carbon source to *P. aeruginosa* that can influence its carbon metabolism, elicit global stress response, and lead to intracellular metal accumulation, improving its competitiveness against other microbes. Furthermore, phenotypes associated with malonate-utilization are not specific to lab strains of *P. aeruginosa*, but also to clinical strains that warrant further investigation.

## Material and Methods

### Bacterial growth and strains

Bacterial strains used in this study are described in Tables 2 and 3. We used the strain PA14 and its isogenic *mariner* transposon mutants (**Supplementary Table 1**) for most of the experiments. Bacterial strains were routinely grown overnight in Luria-Bertani (LB) broth. When needed, antibiotics were added at the following concentrations: 50 μg/mL of ampicillin and 15 μg/mL of gentamicin.

For analysis of the effect of malonate as a sole carbon source on the growth and virulence of PA14, we used the established M9 minimal medium (6.0 g Na2HPO4, 3.0 g KH2PO4, 0.5 g NaCl, 1.0 g NH4Cl per L supplemented with 0.2 mM CaCl_2_ and 2 mM MgSO_4_) (Fisher Scientific) as a basal medium [75]. No iron source was added to the M9 minimal medium. For the control medium, we modified the M9 by adding 1% glycerol (v/v; 110 mM) as a sole carbon source (GM9). We previously utilized this concentration of glycerol as a carbon source in analyzing the regulation of *P. aeruginosa* virulence genes [14]. For M9 media containing malonate as a sole carbon source (MM9), we added 40 mM malonate (or 100 mM malonate if explicitly mentioned), as sodium malonate dibasic (MilliporeSigma, St. Louis, MO). For analysis of gene expression and virulence factor production, PA14 was routinely grown in GM9 and MM9 for 16 hours.

### RNA extraction

PA14 in MM9 and GM9 media was grown overnight for 16 hours at 37°C in shaking condition. The overnight grown culture was then centrifuged at 5,000 rpm speed for 10 min. After discarding the supernatant, bacterial pellets were lysed by the addition of lysozyme and proteinase K for 15 min at room temperature. RNA was extracted using the RNeasy Mini Kit (Qiagen) according to the manufacturer’s protocol. RNA solution was digested with the RNase-free DNase set (Qiagen), followed by on-column DNase digestion to eliminate any remaining traces of genomic DNA. The purified RNA was quantified using a NanoDrop spectrophotometer (NanoDrop Technologies, Wilmington, DE). The samples were then sent to Genewiz for library prep and Illumina HiSeq. Only samples with an RNA integration number greater than 8.0 were used for cDNA library preparation.

### Transcriptomic (RNA-seq) analysis

RNA-seq data were analyzed using Rockhopper software implementing reference-based transcript assembly with UCBPP-PA14 as a reference genome followed by calculating the fold change for the transcripts at each growth condition [20]. NCBI Reference Sequence NC_008463.1 was used. Datasets were normalized using upper quartile normalization, then transcript abundance was quantified using reads assigned per the kilobase of target per million mapped reads normalization method. The selection criteria for differential expression required genes to have a fold change of ≥ 1.5 and a Q value of ≤ 0.05 to be considered significant. The Q value was obtained by adjusting the P value using the Benjamini–Hochberg procedure.

### Real-Time Quantitative Reverse Transcription PCR (qRT-PCR)

One μL of RNA was used for cDNA synthesis. For qRT-PCR, equal amounts of cDNA were mixed with iQ SYBR Green Supermix (Bio-Rad, Hercules, CA) together with 2 μM of specific primers for each gene examined. Amplification and detection were performed using CFX96 Deep Well Real-Time PCR System (Bio-Rad) and analysis of gene expression was done using CFX Manager 3.1 software (Bio-Rad). Each experiment consisted of three biological replicates analyzed in triplicate. Quantity of cDNA in the samples was normalized using the 16S ribosomal RNA gene *PA14_08570*. A list of primers used in this study are mentioned in **Supplementary Table 2**.

### Calculation of the aggregation index

The aggregation index was calculated as the ratio of post-sonication OD_600_ to pre-sonication OD_600_ of overnight cultures [76].

### Intracellular metal ions measurement using ICP-MS

Samples were prepared for ICP-MS following an established protocol with slight modifications [56]. Bacterial cells were cultured overnight in Chelex-treated MM9 and GM9 for 16 hours in the presence of a cocktail of essential metal ions add to the media (Mg 1mM, Mn 25 μM, Ca 100 μM, Fe 5 μM, Zn 25 μM, Co 0.1 μM, and Cu 0.1 μM). These cultures were pelleted and washed in Chelex-treated 500 μL phosphate-buffered saline (PBS). Next, the pellet was suspended in 1 mL metal-free water (Chelex treated) and sonicated at 100% amplitude for 30 seconds each. The probe was washed with Chelex-treated water and the control solution used was sonicated water. The probe was wiped and rinsed between samples as thoroughly as possible using water fresh from our filtration system. We sonicated 1 water sample between every 2 samples to account for metal fluctuations that may occur over the sonication process. Samples were normalized to equal protein concentration and 100 μL of each sample was digested by adding 1 mL 50% HNO3 (Optima grade; Fisher) followed by overnight incubation at 50°C in a metal-free 15-ml conical tube. After digestion, samples were diluted to a 20-ml final volume in Milli-Q water and submitted for inductively coupled plasma mass spectrometry (ICP-MS) analysis. Levels of Ag, Al, As, B, Be, Cd, Co, Cr, Cs, Cu, Fe, Ga, K, Li, Mg, Mn, Na, Ni, Pb, Rb, Se, Sr, Ti, U, V, and Zn were measured. Quality analysis and quality control included Rh-103 and In-115 as internal standards as well as blanks and continuing calibration checks which were run every 10 samples. Calibration standards were prepared in 50 mL polypropylene tubes with 2% HNO_3_ to match the sample matrix. Detection limits for the elements quantified were Li = Cu = 0.01 ppb, Be = 0.0006 ppb, Mg = Al = 0.47 ppb, V = 0.04 ppb, Cr = Mn = Cs = Rb = U = 0.001 ppb, Fe = 0.04 ppb, Co = 0.005 ppb, Ni = Sr = 0.02 ppb, Ga = 0.002, Zn = 0.11 ppb). Ion levels were normalized to the endogenous ion levels of the control samples.

### Co-culture in MM9 and GM9 media

Prior to inoculation of cultures into MM9 and GM9 growth medium, the laboratory reference strain of *P. aeruginosa* UCBPP-PA14 was cultured in LB and the wild-type strain of *S. aureus*, USA300 JE2 was cultured in Tryptic soy broth (TSB). Overnight cultures were washed thrice with filter-sterilized PBS to remove media contamination from either LB or TSB. After a final wash with M9, cells were normalized to an OD_600_ of 1.0 (culture density of ~ 10^8^). Normalized cells were then suspended in MM9 and GM9 as a monoculture or co-culture. Cells were then incubated as monocultures or co-cultures for 24 hours at 37°C under shaking condition. Following incubation, bacterial cells were diluted in sterile PBS and plated on selective media, *P. aeruginosa* monocultures were plated on Pseudomonas Isolation Agar (PIA) plates, *S. aureus* monocultures were plated on Mannitol Salt Agar (MSA) plates, and co-cultures were plated on both plates to observe differences in microbial growth.

### Antibiotic susceptibility testing for bacterial strains

Minimum inhibitory concentration (MICs) of kanamycin and norfloxacin against wild-type and clinical isolates of *P. aeruginosa* was determined in both MM9 and GM9 using broth microdilution method [77]. Overnight cultures were adjusted to an optical density at 600 nm of 1.0 in 1 M9 and diluted to ~ 5 10^5^ colony-forming units (CFU) per ml. Both kanamycin and norfloxacin were diluted in 1 M9, then added to 96-well plates containing bacterial inoculum in either MM9 or GM9. Cells without any drug were also incubated as a growth control. Distilled water and glacial acetic acid were added as vehicle controls for kanamycin and norfloxacin, respectively, as the initial drug stocks were prepared. Plates were incubated at 37°C for 24 hours at static condition. Bacterial growth was assessed using 20μL of 0.2 mg/mL resazurin solutions in each well. Following another 12 hours of incubation with resazurin, the MIC was determined visually by observing the transition from blue (non-viable) to pink (viable) color. We standardized the MIC data in MM9 against GM9 and represented the results in a heatmap for comparative analysis. A value of 1, denoted by a yellow color in the heatmap, indicated that a strain remained unaffected by the action of any tested antibiotic in both media. Increasing tolerance to a specific antibiotic in MM9 was represented by shades of light to deep green, while greater susceptibility was depicted by shades of light to deep purple.

### Statistical Analysis

Statistical analyses and graphics plotting were performed using R 4.0.5 and GraphPad Prism 9.0 (GraphPad Software, Inc., San Diego, CA). Unpaired t-test was used to determine statistical significance (unless otherwise stated).

## Figures and Tables

**Table 1:** Clinical strains used in this study andtheir phenotypes.

Strain	Clinical phenotype
CF MUC 05	Mucoid
CF MUC 07	Mucoid
CFMUC 13	Mucoid
CFMUC 16	Mucoid
CFMUC 19	Mucoid
CF MUC 20	Mucoid
CF MUC 100	Mucoid
CF MUC 135	Mucoid
CF MUC 148	Mucoid
CF MUC 181	Mucoid
CF MUC 221	Mucoid
CF MUC 258	Mucoid
CF MUC 264	Mucoid
CF MUC 294	Mucoid
CF MDR 490	Multidrug-resistant
CF MDR 642	Multidrug-resistant
CF MDR 666	Multidrug-resistant
CF MDR 672	Multidrug-resistant
CF MDR 674	Multidrug-resistant
CF MDR 2350	Multidrug-resistant
CF MDR 2539	Multidrug-resistant
CF MDR 2540	Multidrug-resistant
CF MDR 2577	Multidrug-resistant
CF MDR 2588	Multidrug-resistant

**Table 2:** Description of lab strains used in this study.

Strain	Description	Reference
PA14	*P aeruginosa* strain UCBPP-PA14; prototrophic; clinical isolate from burn patient	[78]
PAK	*P aeruginosa* strain K; hyper piliated; sensitivity to Pf1 phage; prototrophic	[79, 80]
PAO1	*P aeruginosa* strain; prototrophic; clinical isolate from a wound	[81]
JE2	*S. aureus* MRSA strain USA300 LAC cured of plasmids	[82]

**Table 3:** MIC of Kanamycin (KAN) and norfloxacin (NOR) against wild-type and clinical isolates of *P. aeruginosa* in MM9 and GM9 media. ng: no growth,ng-aa: no growth with glacial acetic acid as vehicle control.

Strain	MIC (μg/ml)			
	Kanamycin		Norfloxacin	
	GM9	MM9	GM9	MM9
PA14	150–300	300–600	0.78–0.39	0.09–0.19
PAO1	75–150	300	6.25–12.5	1.56–3.12
PAK	150–300	600–1200	0.19	0.09
CF MUC 5	600	600–1200	1.56–3.12	0.09–0.78
CF MUC 7	1200	ng	ng-aa	ng
CF MUC13	1200–>1200	600–>1200	ng-aa-0.78	ng-0.09
CF MUC16	>1200	>1200	25–100	6.25
CF MUC100	>1200	600–>1200	0.39–>100	0.19–>100
CF MUC135	300–>1200	300–>1200	0.39–6.25	0.09–3.12
CF MUC 148	300–600	600–1200	1.56	0.19–0.39
CF MUC 181	300–600	600–1200	1.56	0.19–0.39
CF MUC 264	>1200	600–>1200	6.25–12.5	0.39–6.25
CF MUC 294	>1200	>1200	12.5	1.56
CF MDR 672	300	600	0.19–0.39	0.09
CF MDR 674	600	1200	0.39–0.78	0.09
CF MDR 2539	300–600	600–1200	1.56	0.39–0.78
CF MDR 2540	600	600–1200	0.19–0.39	0.09–0.39
CF MUC 258	ng	ng	ng	ng
CF MDR 2588	300	300–600	0.39–0.78	0.09–0.19

**Table 4:** Differentially expressed genes in PA14 grown in MM9 in comparison with GM9. Listed genes are relatedto metal stress response.

Gene	Locus	Product	Expression in MM9 relative to GM9
	PA14_18810	hypothetical protein	82.2
	PA14_18820	hypothetical protein	168.9
	PA14_18830	adenylosuccinate lyase	47.1
	PA14_18850	adenylosuccinate lyase	151.2
	PA14_18860	hypothetical protein	68.2
	PA14_18800	hypothetical protein (copper chaperone CopZ domain)	7.2
	PA14_18070	periplasmic metal-binding protein (copper chaperone CopZ domain)	46.8
	PA14_18760	RND efflux membrane fusion protein (MexP homolog)	21.7
	PA14_18780	RND efflux transporter (MexQ homolog)	8.3
	PA14_18790	outer membrane efflux protein (OpmE homolog)	16.8
	PA14_13170	metal transporting P-type ATPase (CopA1/CueA homolog)	21.3
opmD	PA14_09500	outer membrane protein	16.5
mexI	PA14_09520	RND efflux transporter	18.6
mexH	PA14_09530	RND efflux membrane fusion protein	26.2
mexG	PA14_09540	hypothetical protein	32.5
	PA14_45970	cation-transporting P-type ATPase	24.8
	PA14_61330	magnesium transporter, MgtC family	2.4
	PA14_33130	cation-transporting P-type ATPase	−1.8
	PA14_44440	cation-transporting P-type ATPase	2.9
	PA14_44430	hypothetical protein	5.2
	PA14_44420	ferredoxin	6.0
	PA14_61000	hypothetical protein	18.9
azu	PA14_65000	azurin	3.7
pvdH	PA14_33500	diaminobutyrate-2-oxoglutarate aminotransferase	−8.5
	PA14_33520	thioesterase	−3.4
	PA14_33510	hypothetical protein	−2.7
pvdG	PA14_33270	protein PvdG	−6.5
pvdL	PA14_33280	peptide synthase	−5.08
	PA14_33730	dipeptidase	−6.02
pvdO	PA14_33710	protein PvdO	−5.1
pvdN	PA14_33720	protein PvdN	−4.9
	PA14_33540	ABC transporter permease	−1.9
	PA14_33560	adhesion protein	−1.6
	PA14_33550	ABC transporter ATP-binding protein	−1.5
	PA14_33610	peptide synthase	−3.6
pvdD	PA14_33650	pyoverdine synthetase D	−3.5
pvdJ	PA14_33630	protein PvdJ	−3.2
pvdA	PA14_33810	L-ornithine N5-oxygenase	−4.9
pvdE	PA14_33690	pyoverdine biosynthesis protein PvdE	−4.9
fpvA	PA14_33680	ferripyoverdine receptor	−3.5
	PA14_33770	hypothetical protein	−7.8
	PA14_33750	outer membrane protein	−6.4
	PA14_33760	ABC transporter ATP-binding protein/permease	−6.3

**Table 5:** Differentially expressed genes in PA14 grown in MM9 in comparison with GM9. Listed genes are relatedto oxidative stress response.

Gene	Locus	Product	Expression in MM9 relative to GM9
katA	PA14_09150	catalase	54.5
katB	PA14_61040	catalase	34.4
katE	PA14_36810	hydroperoxidase II	−3.1
ahpB	PA14_53300	alkyl hydroperoxide reductase	171.1
ahpC	PA14_01710	alkyl hydroperoxide reductase	16.6
ahpF	PA14_01720	alkyl hydroperoxide reductase	41.1
	PA14_18690	peroxidase	4.8
ohr	PA14_27220	organic hydroperoxide resistance protein	2.0
	PA14_27520	glutathione peroxidase	1.5
trxB2	PA14_53290	thioredoxin reductase 2	46.0
ccpR	PA14_60700	cytochrome c551 peroxidase	36.8
	PA14_61020	hypothetical protein (Ankyrin repeat domain)	11.1
	PA14_21530	ankyrin domain-containing protein	112.2
	PA14_22320	hypothetical protein	184.6
	PA14_03090	hypothetical protein (Two tandem repeats of the cystathionine beta-synthase (CBS pair) domain)	24.7
phhA	PA14_52990	phenylalanine 4-monooxygenase	2.8
hpd	PA14_53070	4-hydroxyphenylpyruvate dioxygenase	6.1
hatE	PA14_57830	hypothetical protein	1.8
hatC	PA14_57850	hypothetical protein	1.6
hatB	PA14_57870	ABC transporter permease	1.6

**Table 6:** Differentially expressed genes in PA14 grown in MM9 in comparison with GM9. Listed genes are relatedto carbon starvation and anaerobic stress response.

Gene	Locus	Product	Expression in MM9 relative to GM9
rpoS	PA14_17480	RNA polymerase sigma factor RpoS	3.5
psrA	PA14_25180	transcriptional regulator PsrA	1.6
ackA	PA14_53470	acetate kinase	2.9
pta	PA14_53450	hypothetical protein	4.5
	PA14_01310	cytochrome C oxidase assembly protein	5.7
coxA	PA14_01300	cytochrome c oxidase subunit I	5.5
coIII	PA14_01320	cytochrome c oxidase subunit III	4.3
coxB	PA14_01290	cytochrome c oxidase subunit II	2.3
nuoK	PA14_29890	NADH dehydrogenase subunit K	4.3
nuoG	PA14_29940	NADH dehydrogenase subunit G	4.0
nuoD	PA14_29990	bifunctional NADH:ubiquinone oxidoreductase subunit C/D	3.9
nuoA	PA14_30020	NADH dehydrogenase subunit A	3.7
nuoB	PA14_30010	NADH dehydrogenase subunit B	3.6
nuoJ	PA14_29900	NADH dehydrogenase subunit J	3.3
nuoI	PA14_29920	NADH dehydrogenase subunit I	3.5
nuoF	PA14_29970	NADH dehydrogenase I subunit F	3.4
nuoH	PA14_29930	NADH dehydrogenase subunit H	3.1
nuoE	PA14_29980	NADH dehydrogenase subunit E	2.9
nuoL	PA14_29880	NADH dehydrogenase subunit L	2.9
nuoM	PA14_29860	NADH dehydrogenase subunit M	2.5
nuoN	PA14_29850	NADH dehydrogenase subunit N	2.2
	PA14_69090	hypothetical protein	4.3
	PA14_69070	ABC transporter ATP-binding protein/permease	2.8
	PA14_69060	ABC transporter permease	1.9
arcC	PA14_68350	carbamate kinase	3.7
arcB	PA14_68340	ornithine carbamoyltransferase	4.5
arcA	PA14_68330	arginine deiminase	5.2
arcD	PA14_68300	arginine/ornithine antiporter	18.2
oprG	PA14_11270	outer membrane protein OprG precursor	31.0
dnr	PA14_06870	transcriptional regulator Dnr	9.0
nirG	PA14_06690	transcriptional regulator	6.0
nirL	PA14_06700	heme d1 biosynthesis protein NirL	3.0
nirC	PA14_06730	c-type cytochrome	5.0
nirM	PA14_06740	cytochrome c-551	5.0
nirS	PA14_06750	nitrite reductase	11.0
nirD	PA14_41540	assimilatory nitrite reductase small subunit	2.0
nirJ	PA14_06670	heme d1 biosynthesis protein NirJ	3.7
nirH	PA14_06680	hypothetical protein	5.5
nirE	PA14_06660	uroporphyrin-III c-methyltransferase	4.5
nirF	PA14_06720	heme d1 biosynthesis protein NirF	4.5
nirN	PA14_06650	c-type cytochrome	2.0
napB	PA14_49260	cytochrome c-type protein NapB precursor	3.5
napA	PA14_49250	nitrate reductase catalytic subunit	2.8
napC	PA14_49270	cytochrome c-type protein NapC	2.7
napE	PA14_49210	periplasmic nitrate reductase NapE	2.1
napF	PA14_49220	ferredoxin protein NapF	2.0
narKI	PA14_13750	nitrite extrusion protein 1	2.5
narL	PA14_13730	transcriptional regulator NarL	2.1
narJ	PA14_13810	respiratory nitrate reductase delta chain	1.5
norC	PA14_06810	nitric-oxide reductase subunit C	4.5
fhpR	PA14_29620	anaerobic nitric oxide reductase transcriptional regulator	2.8
fhp	PA14_29640	nitric oxide dioxygenase	1.5
hcnA	PA14_36330	hydrogen cyanide synthase HcnA	11.5
hcnB	PA14_36320	hydrogen cyanide synthase HcnB	12.2
hcnC	PA14_36310	hydrogen cyanide synthase HcnC	15.5
sicX	PA14_46160	Small RNA	5.8
pqsA	PA14_51430	coenzyme A ligase	2.8
pqsE	PA14_51380	quinolone signal response protein	3.6
pqsC	PA14_51410	PqsC	4.4
pqsD	PA14_51390	3-oxoacyl-ACP synthase	4.8
pqsB	PA14_51420	PqsB	5.0
phnA	PA14_51360	anthranilate synthase component I	3.0
phnB	PA14_51350	anthranilate synthase component II	2.6
phzH	PA14_00640	potential phenazine-modifying enzyme	3.3
phzS	PA14_09400	hypothetical protein	20.4
phzGI	PA14_09410	pyrodoxamine 5'-phosphate oxidase	3.8
phzDI	PA14_09450	phenazine biosynthesis protein PhzD	2.0
phzCI	PA14_09460	phenazine biosynthesis protein PhzC	24.2
phzBI	PA14_09470	phenazine biosynthesis protein	109.5
phzAI	PA14_09480	phenazine biosynthesis protein	25.4
phzM	PA14_09490	phenazine-specific methyltransferase	29.6
phzA2	PA14_39970	phenazine biosynthesis protein	−10.3
phzB2	PA14_39960	phenazine biosynthesis protein	−4.1
phzG2	PA14_39880	pyridoxamine 5'-phosphate oxidase	−2.3
phzC2	PA14_39945	phenazine biosynthesis protein PhzC	−2.3
	PA14_10550	sulfite reductase	16.7
	PA14_10560	hypothetical protein	20.0
	PA14_30430	thiosulfate sulfurtransferase (Rhodanese homology domain)	1.8
mhr	PA14_42860	hypothetical protein	27.0
pumA	PA14_35160	hypothetical protein	23.4
	PA14_44340	cbb3-type cytochrome c oxidase subunit I	12.3
	PA14_44350	cbb3-type cytochrome c oxidase subunit II	20.6
uspO	PA14_66460	hypothetical protein (Universal stress protein family domain)	20.0
uspK	PA14_21220	hypothetical protein (Universal stress protein family domain)	34.9
uspN	PA14_56590	hypothetical protein (Universal stress protein family domain)	27.8
uspL	PA14_41440	hypothetical protein (Universal stress protein family domain)	5.4
uspM	PA14_56220	hypothetical protein (Universal stress protein family domain)	5.2

**Table 7:** Differentially expressed genes in PA14 grown in MM9 in comparison with GM9. Listed genes are related to temperature, osmolarity, and pH stress responses.

Gene	Locus	Product	Expression in MM9 relative to GM9
ibpA	PA14_23680	heat-shock protein IbpA	2.9
htpX	PA14_27480	heat shock protein HtpX (membrane protease)	8.2
htpG	PA14_43850	heat shock protein 90	5.4
grpE	PA14_62990	heat shock protein GrpE	6.1
	PA14_05960	cold-shock protein	−4.0
capB	PA14_21760	cold acclimation protein B	−5.1
ompR	PA14_68700	osmolarity response regulator	2.2
	PA14_72930	hypothetical protein (Predicted lipid-binding transport protein, Tim44 family)	6.2
	PA14_30410	hypothetical protein (YccA-like protein)	8.1

## Data Availability

The raw sequencing data of each sample have been deposited under BioProject accession number PRJNA862157 in the National Center for Biotechnology Information (NCBI) BioProject database.
